# Shotgun Proteomics Links Proteoglycan-4^+^ Extracellular Vesicles to Cognitive Protection in Amyotrophic Lateral Sclerosis

**DOI:** 10.3390/biom14060727

**Published:** 2024-06-19

**Authors:** Beatrice Vilardo, Fabiola De Marchi, Davide Raineri, Marcello Manfredi, Veronica De Giorgis, Alen Bebeti, Lorenza Scotti, Natasa Kustrimovic, Giuseppe Cappellano, Letizia Mazzini, Annalisa Chiocchetti

**Affiliations:** 1 Department of Health Sciences, Interdisciplinary Research Center of Autoimmune Diseases-IRCAD, University of Eastern Piedmont, 28100 Novara, Italy; beatrice.vilardo@uniupo.it (B.V.); davide.raineri@med.uniupo.it (D.R.); natasa.kustrimovic@uniupo.it (N.K.); giuseppe.cappellano@med.uniupo.it (G.C.); 2 Center for Translational Research on Autoimmune and Allergic Diseases, University of Eastern Piedmont, 28100 Novara, Italy; marcello.manfredi@med.uniupo.it (M.M.); veronica.degiorgis@uniupo.it (V.D.G.); 3 Department of Neurology and ALS Center, University Hospital “Maggiore della Carità”, 28100 Novara, Italy; fabiola.demarchi@uniupo.it (F.D.M.); alen.bebeti@gmail.com (A.B.); letizia.mazzini@uniupo.it (L.M.); 4 Department of Translational Medicine, University of Eastern Piedmont, 28100 Novara, Italy; lorenza.scotti@uniupo.it

**Keywords:** proteomics, extracellular vesicles (EVs), amyotrophic lateral sclerosis (ALS), proteoglycan 4 (PRG-4), cognition, cognitive impairment

## Abstract

Amyotrophic lateral sclerosis (ALS) is a progressive neurodegenerative disorder lacking reliable biomarkers for early diagnosis and disease progression monitoring. This study aimed to identify the novel biomarkers in plasmatic extracellular vesicles (EVs) isolated from ALS patients and healthy controls (HCs). A total of 61 ALS patients and 30 age-matched HCs were enrolled in the study and the protein content of circulating EVs was analyzed by shotgun proteomics. The study was divided into a discovery phase (involving 12 ALS and 12 HC patients) and a validation one (involving 49 ALS and 20 HC patients). In the discovery phase, more than 300 proteins were identified, with 32 proteins showing differential regulation in ALS patients compared to HCs. In the validation phase, over 400 proteins were identified, with 20 demonstrating differential regulation in ALS patients compared to HCs. Notably, seven proteins were found to be common to both phases, all of which were significantly upregulated in EVs from ALS patients. Most of them have previously been linked to ALS since they have been detected in the serum or cerebrospinal fluid of ALS patients. Among them, proteoglycan (PRG)-4, also known as lubricin, was of particular interest since it was significantly increased in ALS patients with normal cognitive and motor functions. This study highlights the significance of EVs as a promising avenue for biomarker discovery in ALS. Moreover, it sheds light on the unexpected role of PRG-4 in relation to cognitive status in ALS patients.

## 1. Introduction

Amyotrophic lateral sclerosis (ALS) is a fatal progressive neurodegenerative disease (NDD) marked by the gradual deterioration and demise of both upper motor neurons (UMNs) and lower motor neurons (LMNs), situated in the brain and spinal cord [1]. This degeneration leads to progressive weakness of voluntary skeletal muscles involved in limb movement, swallowing, speaking, and respiratory function [2]. Approximately 90% of cases are classified as sporadic ALS (sALS) while the remaining 10% are classified as familial ALS (fALS). sALS emerges from a combination of genetic and behavioral factors and exposure to environmental risks, while fALS is associated with a genetic predisposition and specific inheritance pattern [3]. Both forms of the disease present remarkably similar symptoms and share numerous pathogenic pathways. These include factors of both genetic and environmental origin contributing to cellular dysfunction, such as excitotoxicity [4], endoplasmic reticulum stress [5], abnormal protein aggregation [6], autophagy dysregulation [7], prion-like propagation [8], and secretion of neurotoxic vesicles by surrounding cells such as astrocytes and muscle cells [9]. Furthermore, mitochondrial disorganization and dysfunction leading to oxidative stress [10], as well as alteration of RNA processing, are implicated in the disease progression. Ultimately, these pathophysiological processes, either individually or in combination, result in the alternation of the electro-physiological properties of the motor neuron, ultimately resulting in their death. 

Understanding these properties is crucial for comprehending the pathophysiology of ALS. Among electrophysiological changes, it has been shown that increased persistent sodium currents in motoneurons from mutant superoxide dismutase-1 (SOD1) mice may affect excitability and may contribute to motoneuron degeneration [11]. Moreover, sensory pathway abnormalities in ALS patients suggest a pattern of axonal loss primarily in large-caliber myelinated fibers [12], implicating sensory pathway involvement in ALS pathogenesis. Furthermore, general sensory system abnormalities are associated with the degeneration of motor neuron and dorsal root ganglion cells [13]. Advanced non-invasive techniques, such as surface electromyography-based motor unit number estimation, show promise for early detection of ALS-specific alterations [14]. Additionally, ALS patients may exhibit demyelinating features in peripheral nerves, suggesting the involvement of both axonal degeneration and temporal demyelination [15]. Thus, understanding these electrophysiological properties is essential for unraveling the complex neurodegenerative processes underlying ALS. 

From a clinical standpoint, ALS has been recognized as a complex syndrome with a great phenotypic variability. Behavioral and cognitive changes are observed in approximately 35 to 50% of ALS patients. Importantly, these patients typically experience a worse prognosis, with shorter survival. Additionally, approximately 15% of ALS patients meet the diagnostic criteria for frontotemporal dementia (FTD). The presence of cognitive and behavioral impairment support the concept of ALS as a neurodegenerative disease with a broad spectrum of manifestations, sharing a continuum with FTD [16].

ALS is associated with various risk factors, including smoking, chemical exposure, and genetic predisposition. Over 30 genes are implicated in ALS, with SOD1, TAR DNA-binding protein-43 (TDP-43), fused in sarcoma (FUS), and chromosome 9 open reading frame 72 *(C9ORF72*) being among the most commonly mutated genes. Recent research highlights the significance of the kinesin family member 5A (KIF5A) gene, involved in neuronal function, in ALS pathogenesis. Mutations in KIF5A, particularly in its cargo-binding tail domain, are linked to ALS, leading to motor neuron damage [17]. While these genetic factors account for a significant portion of familial ALS cases, with SOD1 and C9ORF72 mutations being primary culprits [18], they only explain a subset of ALS cases. Many patients with ALS do not have identifiable genetic mutations, making it difficult to rely only on genetic biomarkers for diagnosis or prognosis. While genetic biomarkers are valuable for understanding the hereditary aspects of ALS and for identifying potential therapeutic targets, they need to be complemented with other types of biomarkers and diagnostic tools. Recent research has concentrated on exploring biomarkers beyond genetic markers to improve diagnosis and treatment of ALS. Mass spectrometry-based proteomics has emerged as a promising approach for identifying biomarkers for ALS [19]. Identifying these markers should be a crucial goal for researchers, as it facilitates the design of clinical trials and the evaluation of new treatments [20]. 

In this regard, extracellular vesicles (EVs) have garnered significant attention in the field of ALS and other NDDs as potential biomarkers, due to their ability to act as carriers of misfolded proteins like SOD-1, TDP-43, and FUS, enabling their propagation between cells [21]. EVs are spherical particles released by diverse cell types under both physiological and pathological conditions. They play a vital role in the intricate mechanism of cell-to-cell communication, serving as key mediators for the transmission and dissemination of pathological proteins to recipient cells [22]. Furthermore, the significance of EVs as potential biomarkers is further underscored by their ability to be isolated from a range of bodily fluids, including blood, urine, saliva, and cerebrospinal fluid. Among these fluids, blood emerges as the most pertinent reservoir of EVs. The impact of EVs is largely determined by their surface molecules and cargo, which comprise proteins, lipids, nucleic acids like DNA and RNA, and metabolites sourced from the originating cell [23]. Characterizing the cargo of EVs through proteomics, transcriptomics, and metabolomics provides a source of biomarkers accessible through non-invasive liquid biopsies [24]. Among these approaches, proteomic profiling has been shown to be particularly promising [25,26]. 

In the present study, by applying proteomic analysis of plasma-derived EVs isolated from ALS patients and HCs we identified a significant number of proteins that were differentially regulated in EVs derived from ALS patients compared to HCs in the discovery (>300) and validation (>400) phases of the study. Notably, seven proteins were common for both cohorts, and found to be consistently upregulated in EVs from ALS patients. Among these proteins, PRG-4 exhibited the highest fold-change and demonstrated a correlation with clinical outcomes. In particular, we observed that PRG-4 levels were notably decreased in ALS patients with impaired cognitive function, as assessed at the time of ALS diagnosis. This finding suggests a potential association between PRG-4 expression levels in EVs and cognitive status among ALS patients.

## 2. Materials and Methods

### 2.1. Patients

Peripheral venous blood samples were collected from ALS patients (n = 61) and HCs (n = 30). Patients were recruited at the Tertiary ALS Center (CRESLA) at the Neurology Clinic of the “Maggiore della Carità” University Hospital in Novara, Italy, in a period from January 2020 to October 2021. Each participant included in the study provided written informed consent prior to the involvement. The study was performed according to the relevant ethical guidelines for research on humans, conducted in accordance with the declaration of Helsinki principles, and approved by the Maggiore della Carità Hospital Ethical Committee (Protocol n. CE n. 184/20). At the baseline (T0), all patients underwent clinical and neurological examination, including the compilation of the ALS Functional Rating Scale—Revised (ALSFRS-R) score, pulmonary function test with the measurement of forced vital capacity (FVC) percentage, and evaluation of the body mass index (BMI). The patients, in collaboration with the multidisciplinary team at CRESLA, were longitudinally followed throughout the course of their disease. Data were collected at T0, and then, at three-month intervals throughout the entirety of the disease’s duration. Monthly changes in ALSFRS-R score, FVC percentage, and BMI between the first and last assessments were calculated using the formula

(measurement at the first assessment − measurement at the last available assessment)/(date of the first assessment − date of the last assessment)(1)

Considering the rate of decline of the ALSFRS-R, based on the median delta progression, the patients were divided into “slow progressors” (≤ of median) and “fast progressors” (> of median). Survival time was calculated at the time of death (or tracheostomy); for patients still living, 31 March 2024 was set as the last data entry point. The cognitive status with a complete neuropsychological test battery was evaluated and patients were classified according to the Strong classification (ALS-normal, ALS-Bi, ALS-Ci, ALS-CiBi, ALS-FTD) [27]. 

The inclusion and exclusion criteria were as follows:

Inclusion criteria for patients: age > 18 years and <75 years; patients under the care of the CRESLA at the Neurology Clinic of the “Maggiore della Carità” University Hospital in Novara; disease duration not exceeding 24 months and not exceeding 18 months from diagnosis (early/intermediate disease stage); patient has capacity and willingness to provide informed consent or the presence of a legally authorized representative willing to give this. Informed consent was obtained by neurologists at the CRESLA of the Neurology Clinic and stored in the patient’s medical record. 

Exclusion criteria for patients: presence of non-neurodegenerative neurological disorders; presence of other comorbidities that could introduce bias (e.g., autoimmune diseases, diabetes mellitus); insufficient clinical/phenotypic disease data.

In addition, the following information was collected for each patient. Demographic characteristics: gender, date and place of birth, and education level; anamnestic data including concurrent illnesses (heart diseases, hypertension, thyroid disorders, neoplasms, autoimmune diseases, diabetes, or other pathologies), ongoing therapies, exposure to toxins, physical activity, and smoking.

Clinical disease information collected included: onset date and age of symptoms, date and age at diagnosis, type of onset (spinal or bulbar), signs at diagnosis (bulbar, upper limb, lower limb, or involvement of I and/or II motor neuron), ALSFRS-R score, FVC percentage values, and cognitive status (according to Strong’s classification, patients were categorized as normal, ALS-bi, ALS-ci, ALS-bici, FTD).

### 2.2. Quantification of EVs by Nanoparticle Tracking Analysis (NTA)

According to the ISEV 2018 guidelines [28], 1 mL of plasma of ALS patients and HCs was ultracentrifuged at 100,000× *g* at 4 °C for 1 h (Thermo Scientific, Waltham, MA, USA, Sorvall VX + Ultracentrifuge Series) to isolate the pellet enriched in EVs. After the isolation, EV pellets were resuspended in filtered phosphate-buffered saline (PBS) and nanoparticle tracking analysis (NTA) was performed to characterize their dimensions and concentration using the Nanosight NS300 instrument (Malvern, Framingham, MA, USA). Samples were diluted in filtered PBS (1:40) and each sample was recorded for 60 s, with manual monitoring of temperature and the camera level set at 13. Analysis was performed using the NTA v3.3 software (Malvern, Framingham, MA, USA), with the detection threshold set to 5 EVs; concentration was expressed in EVs/mL, while EV size was expressed in nm using the mode value.

### 2.3. Scanning Electron Microscopy (SEM)

A drop of each EV sample was placed on a glass coverslip and fixed with 2.5% glutaraldehyde (Electron Microscopy Sciences, Hatfield, PA, USA) for 20 min at room temperature (RT) to visualize the structure by SEM. The samples were processed through a graded ethanol series (70%, 90%, and 100% ethanol, Merck, Italy). Following this, a drop of hexamethyldisilazane (HMDS, Electron Microscopy Sciences, Hatfield, PA, USA) was applied to the samples and allowed to dry completely. Once dried, the samples were coated with a nanolayer of gold using a sputter coater (DII-29030SCTR Smart Coater, JEOL SpA, Basiglio, Milan, Italy). Images were acquired using a bench scanning electron microscope (SEM, JSM-IT500, JEOL SpA) at a magnification of 30,000×.

### 2.4. Western Blot Analysis 

A total of 20 μg of EV lysates were loaded on 8% SDS-PAGE gel and were transferred into a nitrocellulose membrane. The membrane was blocked for 1 h with 3% BSA and incubated overnight at 4 °C with the following antibodies: anti-CD63 (Invitrogen, diluted 1:500), anti-CD9 (Invitrogen, diluted 1:500), anti-CD81 (Invitrogen, diluted 1:500), calnexin (Invitrogen, diluted 1:5000), anti-apolipoprotein A1 (Merck, diluted 1:1000), and anti-apolipoprotein B (Merck, diluted 1:1000) in 3% of BSA. After incubation with primary antibodies, the membrane was washed with 0.1% tween in tris-buffered saline (TBST) three times for 10 min. Incubation with a secondary antibody was performed at room temperature (RT) with goat anti-mouse IgG secondary antibody HRP conjugated (Invitrogen) for 1 h. The membrane was washed three times with TBST for 15 min, and then, exposed to an ECL chemiluminescence kit (Western Nova 2.0, Cyanagen) in 1:1 ratio and incubated for 2 min. The images were acquired using the Chemidoc (BIO-RAD, Chemidoc imaging system).

### 2.5. Proteomics Analysis 

The EVs were lysed with RIPA buffer (150 mM NaCl, 1% Triton X-100, 0.5% sodium deoxylcholate, 0.1% SDS) and sonicated. Proteins were then precipitated with cold acetone and resuspended. The proteins were then reduced in 25 µL of 100 mM NH4HCO3 with 2.5 μL of 200 mM dithiothreitol (DTT) (Merck) at 60 °C for 45 min and next alkylated with 10 μL 200 mM iodoacetamide (Merck) for 1 h at room temperature (RT) in dark conditions. Excess iodoacetamide was removed by the addition of 200 mM DTT. The digests were dried by speed vacuum, and then, desalted [29]. The digested peptides were analyzed on an Ultimate 3000 RSLC nano coupled directly to an Orbitrap Exploris 480 with a high-field asymmetric waveform ion mobility spectrometry system (FAIMSpro) (all Thermo Fisher Scientific). Samples were injected onto a reversed-phase C18 column (15 cm × 75 µm i.d., Thermo Fisher Scientific) and eluted with a gradient of 6% up to 95% mobile phase B over 80 min by applying a flow rate of 300 nL/min, followed by an equilibration with 6% mobile phase B for 8 min. Mass spectrometry scans were performed in the range of *m*/*z* 375–1200 at a resolution of 120,000 (at *m*/*z* = 200). MS/MS scans were performed choosing a resolution of 15,000; normalized collision energy of 30%; isolation window of 2 *m*/*z*; and dynamic exclusion of 45 s. Two different FAIMS compensation voltages were applied (−45 V and −60 V), with a cycle time of 1.5 s per voltage. The FAIMS was operated in standard resolution mode with a static carrier gas flow of 4.6 L/min. The acquired raw MS data files were processed and analyzed using Proteome Discoverer with Chimerys (v3.0.0.757, Thermo Fisher Scientific). SequestHT was used as a search engine and the following parameters were chosen. Database: Homo sapiens (Uniprot, downloaded on 1 February 2022; enzyme: trypsin; max. missed cleavage sites: 2; static modifications: carbamidomethyl (C); dynamic modifications: oxidation (M); precursor mass tolerance: 10 ppm; fragment mass tolerance: 0.02 Da. Only peptides and proteins with an FDR value < 0.01 were reported. Abundance of identified peptides was determined by label-free quantification (LFQ) using the match between runs. Statistical analyses and *t*-tests were performed on protein abundances using the MetaboAnalyst software (version 5.3) (https://www.metaboanalyst.ca/; accessed on 1 February 2022). Modulated proteins were analyzed through the Database for Annotation, Visualization and Integrated Discovery (DAVID) (version 6.8) (http://david.abcc.ncifcrf.gov/; accessed on 1 February 2022) and IPA (Ingenuity Pathway Analysis, QIAGEN).

### 2.6. Analysis of PRG-4 Expression in EVs of ALS Patients and HCs

To confirm the findings from the proteomic analysis and clinical correlation regarding PRG-4, the levels of PRG-4 in EVs derived from ALS patients (n = 38) and HCs (n = 19) were quantified by ELISA following the manufacturer’s guidelines (R&D Systems, Minneapolis, MN, USA). Two-hundred microliters of serum were ultracentrifuged at 100,000× *g* for 1 h (Thermo Scientific Sorvall VX + Ultracentrifuge Series). The pelleted EVs were resuspended in 200 µL of filtered 1X PBS, and then, diluted 1:25 to perform ELISA.

### 2.7. Statistical Analysis 

The data from the proteomic analysis were analyzed by the GraphPad Prism software v10.2 for comparisons between ALS patients and HCs using the non-parametric Mann–Whitney U test. The level of significance was set at a *p* value < 0.05. In order to detect an association between clinical parameters and proteomic data, the comparison of standardized areas to ensure a uniform scale or reference point for meaningful comparisons was utilized. Specifically, the analysis was limited to subjects exhibiting classic (n = 32) and bulbar (n = 20) phenotypes for the phenotype correlation. For the cognitive behavioral profile correlation, only subjects with a normal (n = 39) or cognitive impairment (n = 20) profile were included to ensure relevance and specificity to the intended investigation. Within each subgroup, median values along with the first and third quartiles of standardized areas were computed. To evaluate group differences, the Mann–Whitney test, considering the non-normal distribution of the areas, was used. In addition, for each comparison the fold change, to provide additional insights into the magnitude of observed differences, was calculated.

## 3. Results

### 3.1. Clinical Characteristics 

The clinical features of the enrolled patients are summarized in Table 1. On average, there was an 11-month interval between the disease onset and its diagnosis. The cohort consisted of 39 males and 22 females. Patients were categorized based on their phenotype at onset (spinal n = 41 and bulbar n = 20). Patients with spinal phenotype were further classified in classic (n = 32), pure/predominant upper motor neuron (PUMN) (n = 6), and respiratory (n = 3) phenotypes. Based on the disease progression patients were additionally stratified into slow (n = 34) and fast progressors (n = 27). Based on cognitive impairment patients were grouped as normal (n = 39) or impaired (n = 20) cognitive status (ALS-Bi, ALS-Ci, ALS-CiBi, ALS-FTD based on Strong classification). To ensure a similar lifestyle and dietary habits, the HC cohort (n = 20) was composed of age-matched cohabitants of the ALS patients.

### 3.2. The Size and Concentration of Plasmatic EVs Are Similar between ALS Patients and HCs

**Figure 1:**
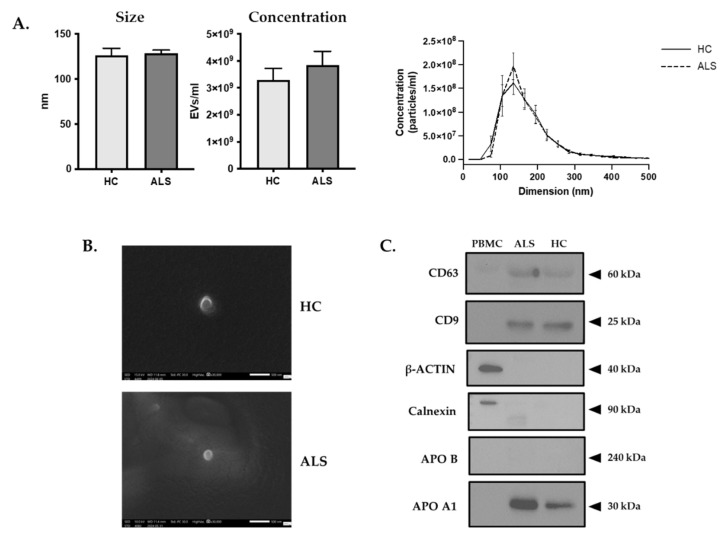
EV characterization by NTA. (**A**) The modal size (nm) of EVs, their concentration in plasma expressed as particles per mL, and the EVs size distribution and concentration of HC compared to ALS patients are shown. (**B**) Representative images of EVs from plasma of HC and ALS patients obtained by SEM (high vacuum; magnification 30,000×; probe current 30). (**C**) Western blot analysis of the EV markers (CD9 and CD63 tetraspanins), cell markers (β-actin and calnexin) and plasma contaminants (APOA1, APOB48/B100) in ALS patients and HCs.

NTA was utilized to determine the size and concentration of EVs in both the HC and ALS patients. Plasmatic EVs from both ALS patients and HCs showed no significant differences in size (*ALS* vs. *HC* average: 126 nm vs. 130 nm) or concentration (*ALS* vs. *HC*: 3.84 × 10^9^ EVs/mL vs. 3.30 × 10^9^ EVs/mL) (Figure 1A). Additionally, no significant correlations were observed between age or gender and EV concentration in either ALS patients or HCs. A trend showing increased amounts of EVs with age was noted in the patient cohort only (Supplementary Figure S1). These findings require confirmation in a larger cohort of patients. 

To further characterize the purified EVs, SEM and western blot analysis of EVs from both ALS patients and HCs was performed. SEM confirmed the NTA results, showing spheroid and cup-shaped EV morphologies, with an average diameter of approximately 135 nm, consistent with the known shape characteristics of EVs and the NTA mean size (Figure 1B). Adhering to the MISEV2018 guidelines, the quality of the purified EVs was further assessed by examining for markers from categories 1 and 2 (CD9, CD63) and non-EV markers from categories 3 and 4 (APO A1, APO B48-100, and calnexin) [28]. Proteins extracted from peripheral blood mononuclear cells (PBMCs) served as positive (calnexin and β-actin) and negative (all the other) controls. Western blot analysis demonstrated the presence of tetraspanins CD9 and CD63 in EVs from both ALS patients and HCs (Figure 1C), with similar expression levels observed between the two groups. These markers were absent in the cell extract. Conversely, calnexin and β-actin were expressed by cells and not by EVs. Apolipoprotein A1 (APO A1), an indicator of high-density lipoprotein (HDL), was enriched in EVs derived from both ALS patients and HCs, whereas the low-density lipoprotein (LDL) marker apolipoprotein B-48/B-100 (APO B48/B100) was scarcely detectable (Figure 1C). 

### 3.3. Proteomics Analysis of EVs

**Figure 2:**
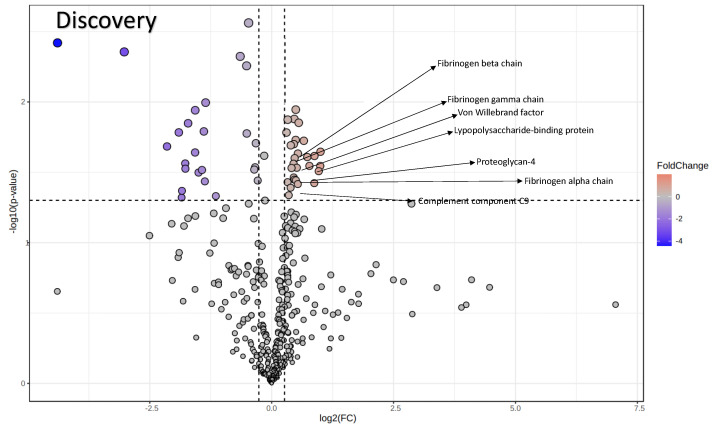
The volcano plot illustrates the significantly differentially abundant proteins identified in discovery phase. The seven proteins that are common across both phases are indicated with arrows. The −log_10_(Benjamini–Hochberg-corrected *p* value) is plotted against the log_2_(fold change).

Plasma represents a good source of circulating EVs, representing an attractive surrogate for biomarker discovery. Upon ultracentrifugation of plasma, EVs from both ALS patients and HCs were collected, lysed, and the protein content was analyzed by shotgun proteomics. We conducted an initial discovery phase including the first 12 enrolled ALS patients and their matched HCs (n = 12). This pilot study allowed us to set the pipeline and to identify and quantify 397 proteins. The statistical analysis performed on protein abundances revealed the presence of 32 differentially expressed EV proteins including fibronectin (fold change (FC) = 1.38), vitamin K-dependent protein S (FC = 1.31), and vitronectin (FC = 0.80), among others (Figure 2, Table 2, and Supplementary Figure S2).

**Figure 3:**
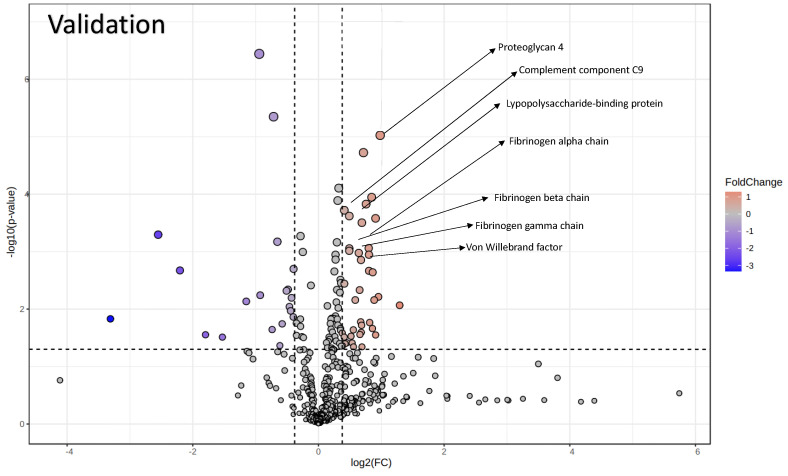
The volcano plot shows the significantly differentially abundant proteins identified during the validation phase. Arrows highlight the seven proteins that are common to both phases. The plot features the −log_10_(Benjamini–Hochberg-corrected *p* value) on the *y*-axis and the log_2_(fold change) on the *x*-axis.

The same EV proteomic analysis was applied to a more abundant validation cohort, which included 49 ALS patients and 20 HCs. A total of 471 proteins was identified, among them some were significantly changed in ALS patients (Figure 3, Table 3, and Supplementary Figure S3).

**Figure 4:**
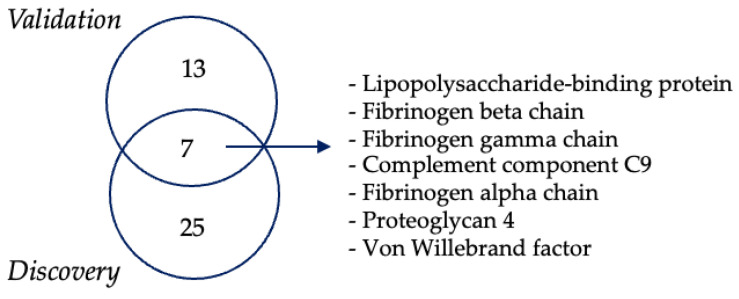
Validation and discovery phases of proteomic analysis revealed seven common upregulated proteins in ALS EVs.

Seven proteins were modulated in both the discovery and the validation cohorts (Figure 4), including fibrinogen α (FIBA), fibrinogen β (FIBB), fibrinogen γ (FIBG), von Willebrand factor (VWF), complement component 9 (C09), proteoglycan 4 (PRG-4), and lipopolysaccharide-binding protein (LBP). 

**Figure 5:**
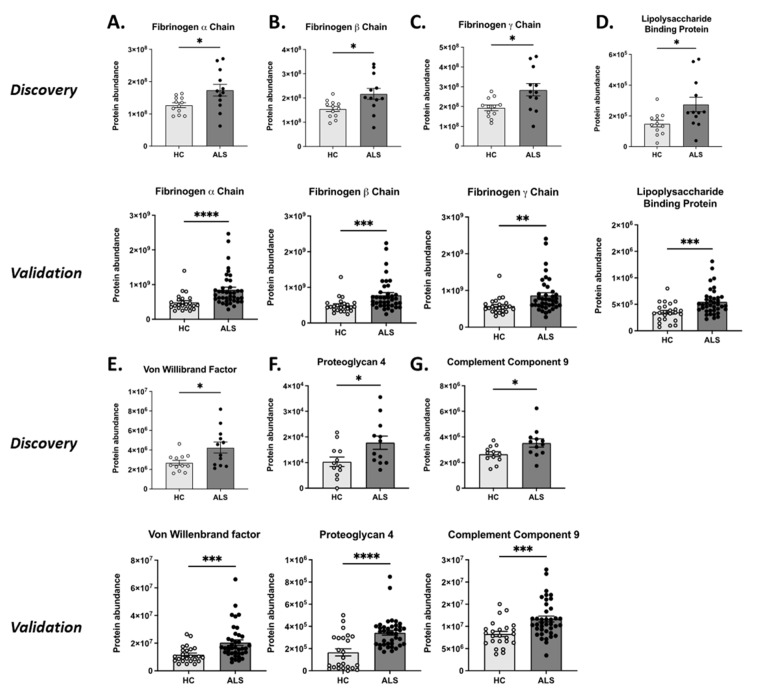
Each graph illustrates the seven proteins identified in both the discovery and validation phases. (**A**) Fibrinogen α (FIBA); (**B**) fibrinogen β (FIBB); (**C**) fibrinogen γ (FIBG); (**D**) von Willebrand Factor (VWF); (**E**) complement component 9 (C09), (**F**) lipopolysaccharide-binding protein (LBP), and (**G**) proteoglycan 4 (PRG-4). For statistical analysis Mann–Whitney test was used: * *p* < 0.05, ** *p* < 0.01, ***p < 0.001, and **** *p* < 0.0001.

These proteins were significantly increased in ALS patients compared to HCs (Figure 5). 

### 3.4. PRG-4-Enriched EVs Are Associated with Normal Cognitive Function in ALS Patients

In our investigation, we explored the potential associations between the seven proteins, previously identified by proteomic analysis, with gender and clinical features, (bulbar or spinal phenotype), cognitive profile (normal or impaired, grouping ALS-ci, ALS-bi, ALS-FTD), and progression rate (fast or slow), as well as non-categorical clinical variables, including ALSFRS-R baseline, and FVC% baseline (Supplementary Tables S1–S4). We found that EVs enriched in C9 protein were inversely correlated with ALSFRS-R. Furthermore, EVs enriched in PRG-4 protein were significantly increased in ALS patients with a normal cognitive profile compared to those with cognitive impairment (Supplementary Table S6). Notably, we found significantly increased levels of PRG-4 between HC and ALS patients with cognitive impairment (ALS-ci) (median HC = 90,713 vs. ALS-ci = 281,055, *p* = 0.0199). These findings suggest a potential association between elevated PRG-4 levels and protection from cognitive impairment in ALS patients. Although a subgroup of HCs showed high PRG-4 levels, no significant correlation with either gender or age was found (Supplementary Figure S4).

### 3.5. ELISA Confirms Increased PRG-4 Expression Levels in EVs of ALS Patients Compared to HCs 

**Figure 6:**
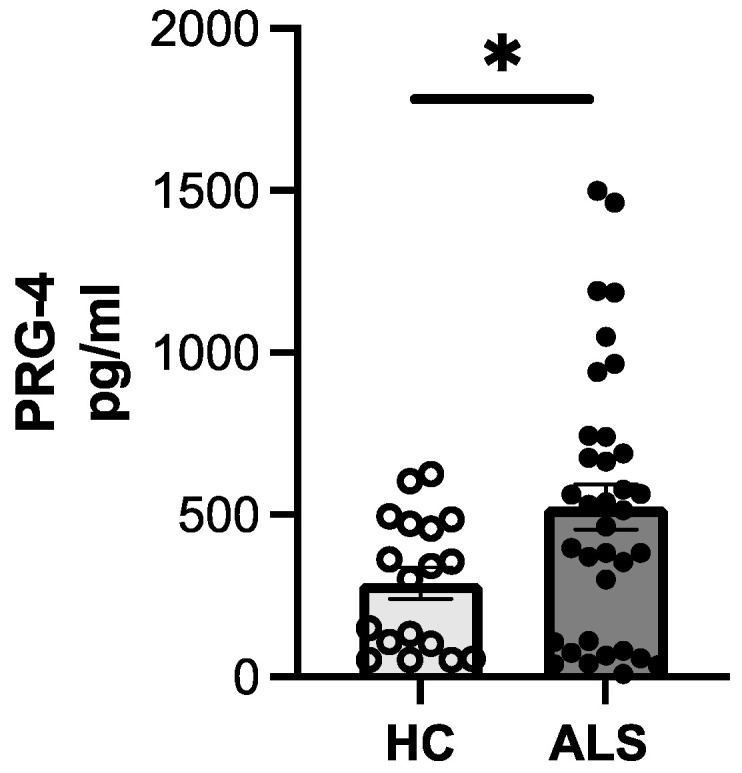
PRG-4 levels evaluated by ELISA in pelleted EVs diluted 1:25. For statistical analysis Mann–Whitney test was used: * *p* < 0.05.

Given the elevated levels of PRG-4-enriched EVs in ALS patients with normal cognitive function, we sought to validate these results by ELISA on EVs collected via ultracentrifugation. Our results confirmed a significant increase in PRG-4 levels in EVs in ALS patients in comparison with HCs (Figure 6), consistent with the findings from the proteomic analysis. Similar results were found when ALS patients were stratified based on cognitive impairment (ALS-ci) and compared to HCs (HC vs. ALS-ci, *p* = 0.0199). 

## 4. Discussion

In this study, we employed a proteomic approach to investigate EVs’ cargo in search of novel diagnostic biomarkers for ALS. Through both the discovery (>300) and validation (>400) phases of the study we identified seven proteins in EVs—FIBA, FIBB, FIBG, C09, VWF, LBP, and PRG-4—that were significantly upregulated in ALS patients in comparison with HCs. Adhering to the MISEV2018 guidelines [28], we evaluated the presence of the tetraspanins (CD9 and CD63), as detailed in Table 3 [28], and non-EV markers (APO A1, APO B48-100, b-actin, and calnexin) by western blotting. Our results demonstrated that EVs from both ALS patients and HCs expressed the EV markers CD63 and CD9, while they were negative for the non-EV markers APOB and calnexin, confirming EV purification from cells. Nevertheless, the finding that EVs from ALS patients and HCs express APO A1 would suggest the presence of soluble proteins, that has been shown many times in the literature [30]. 

It is noteworthy that when working with plasma specimens, distinguishing between lipoprotein particle contaminants and EV-associated lipoproteins can be challenging [30]. 

It has been proposed that soluble proteins can bind to the surface of EVs with two different and non-mutually exclusive mechanisms, involving either a direct or an indirect binding to the EV’s surface. Both can adapt to PRG-4 too. A first hypothesis is based on the fact that PRG-4 contains heavily glycosylated mucin-like domains that are capable of direct binding to the lipid bilayer of EVs through electrostatic interactions, as already demonstrated [31]. A second hypothesis considers an indirect attachment of soluble PRG-4 to EVs through receptors, whether glycosylphosphatidylinositol (GPI)-anchored or transmembrane types, which are known molecular constituents of the EV surface [32,33]. By drawing parallels to the established interaction between PRG-4 and CD44, a transmembrane receptor found on the surface of EVs [33], we speculate that a similar mode of interaction could occur between PRG-4 and GPI-anchored receptors. Notably, EVs have been shown to express CD55, a GPI-anchored protein [34] to which PRG-4 could potentially bind, as demonstrated elsewhere [35]. Among all the identified proteins, PRG-4 showed the highest fold change and it was the only one associated with a clinical outcome. In particular, PRG-4 was decreased in ALS patients with impaired cognitive function, evaluated at the time of diagnosis of ALS.

ALS is characterized by the progressive buildup of pathological proteins such as SOD-1, TDP-43, and FUS, which disrupt neuronal function and ultimately result in neuronal death. Presently, ALS lacks reliable biomarkers that could facilitate diagnosis, prognosis, and assessment of therapeutic effectiveness. In recent years, EVs have emerged as promising candidates for non-invasive liquid biopsies. This interest is primarily driven by the understanding that EVs can transport disease-related signaling molecules. Moreover, advancements in detection techniques enable identification of these disease-associated molecules even at exceedingly low concentrations, despite the highly complex backgrounds such as plasma or serum. EVs have been proposed as a valuable source of biomarkers, particularly in the context of autoimmunity [22], cancer [36], and neurodegenerative diseases [37].

The identified proteins in our study are known to be implicated in inflammatory processes and have been previously associated with ALS, although they are typically detected in either serum or CSF. FIBA, a macromolecular glycoprotein, plays a role in the body’s inflammatory response and tissue healing processes by interacting with various types of nervous system cells, facilitated by its distinctive molecular structure [38]. Keizman et al. reported a significant correlation between ALSFRS-R score and fibrinogen plasmatic levels, which were significantly increased in ALS patients. Other studies have shown a positive correlation between the concentration of FIBB and survival in ALS patients [39]. Specifically, ALS patients who had higher levels of FIBB in their plasma exhibited longer survival periods, a finding consistent across various potential influencing factors like age, gender, respiratory issues, or functional ability [40]. C9 is among the proteins engaged in the complement system, serving as a defense mechanism of the body against pathogens [41]. Karasu et al. observed a cytotoxic effect on oligodendrocytes following complement activation, leading to the formation of C9 and subsequent release of EVs rich in membrane attack complex (MAC) from their surface [42]. Complement activation may contribute to the progressive loss of motor neurons during the advancement of the disease [43]. In our study, we found that ALS patients with a high ALSFRS-R score had less C9-enriched EVs, suggesting their involvement in neurodegeneration. Additionally, an increased expression of activated complement components in both the spinal cord (SC) and motor cortex (MCx) of ALS patients has been confirmed in the literature [43]. 

VWF acts as a stabilizer of the factor eight (FVIII) in the coagulation cascade [44]. Proteomic analysis of CSF from ALS patients revealed enrichment of pathways associated with complement and coagulation cascades along with protein biomarkers that differentiate the rate of progression in ALS. By employing a mathematical model of the CSF proteome, researchers determined that changes in the entropy of the proteome over time serve as a predictive factor for distinguishing between fast progression and slow progression of the disease [45]. 

LBP is primarily produced in hepatocytes, and it is released into the blood during the acute phase of immune response, playing a crucial role in the innate immune response. Zhou et al. reported an increase in LBP levels in CSF from ALS patients in comparison with HCs. These levels correlated with the ALSFRS-R score [46].

PRG-4-enriched EVs were found to be increased in ALS patients compared to HCs. PRG-4, also known as lubricin, is primarily associated with joint health and is not currently recognized for its role in the development or progression of ALS. It is predominantly found in tissues and synovial fluid of the joints, where it functions as a lubricant and protects the joints [47]. The increased levels of PRG-4 in ALS patients may be correlated to the progression of the disease. As ALS advances and voluntary motor movements deteriorate, the physiological integrity of articulation declines. This deterioration could potentially result in an elevated concentration of PRG-4 within EVs. This speculation suggests a possible association between the physiological changes occurring in ALS and the observed increase in PRG-4 levels, highlighting the need for further investigation into the role of PRG-4 in the pathogenesis of ALS. 

Molecules associated with EVs are promising biomarker candidates due to their presence in easily accessible body fluids and their ability to provide insight into the state of the donor cells [48]. This is particularly relevant in neurodegenerative diseases where the target cells are challenging [49]. However, investigating EVs poses challenges due to the complexities of isolating and concentrating EVs out of complex body fluids. To ensure the reliability of our findings, we cross-referenced our data with the EV databases (http://www.microvesicles.org/) and found references to all of the seven identified proteins. Notably, PRG-4-positive EVs, identified via mass spectrometry, have been documented in plasma [50,51], which is in line with our findings. Moreover, PRG-4-positive EVs have been identified in other bodily fluids as well [51,52,53,54]. Moreover, consistent results have been found when EVs were purified using various other methods including filtration, size-exclusion chromatography, sucrose density gradient, and differential centrifugation [51,52,53,54,55,56,57,58]. 

Our findings are in line with Bereman et al., who conducted a proteomics analysis in the plasma of ALS patients, identifying PRG-4 protein as one of the top ten altered proteins [59]. Nevertheless, the precise role of this protein in ALS remains unknown. Indeed, Bennet et al. showed that exogenous administration of recombinant PRG-4 in a periclinal model of traumatic brain injury reduces neuroinflammation by restoring the blood–brain barrier after traumatic brain injury [60]. This suggests that PRG-4 may have potential therapeutic implications beyond its conventional role in joint health.

In ALS, both BBB and the blood–spinal cord barrier (BSCB) become hyperpermeable, enabling a passage for a broader array of molecules, thereby contributing to a more severe and rapidly progressing disease course [61]. The breakdown of the BBB has also emerged as a potential early indicator of human cognitive dysfunction in Alzheimer’s disease [62] and vascular mild cognitive impairment [63], leading us to hypothesize on the correlation observed by us between elevated levels of PRG-4 and cognitive impairment of ALS patients. 

Our findings would suggest PRG-4 EVs could serve as a candidate biomarker for two important aspects of ALS. i) Early diagnosis of ALS: elevated levels of PRG-4 EVs may indicate the onset of ALS, potentially enabling early detection and intervention; ii) monitoring the cognitive-behavioral profile: the correlation between PRG-4 levels and cognitive impairment in ALS patients suggests that PRG-4 EVs could be utilized to monitor cognitive function in ALS patients over time.

Further studies are warranted in order to reveal the connections or shared pathways between ALS and PRG-4, shedding light on novel aspects of ALS pathophysiology. These investigations could reveal novel aspects of ALS pathophysiology and provide valuable insights into disease mechanisms. By elucidating the role of PRG-4 EVs in the ALS process, we may identify new therapeutic targets and develop more effective treatment strategies. Therefore, future research efforts aimed at validating PRG-4 EVs as a biomarker for ALS progression hold promise for advancing our understanding and management of this neurodegenerative disorder.

## 5. Conclusions

As of today, no reliable markers exist for ALS diagnosis, prognosis, and therapeutic assessment. EVs have emerged as potentially relevant biomarkers in liquid biopsies due to their ability to transport disease-related molecules, even in complex biological backgrounds. In our study, proteomic analysis of EVs’ cargo in ALS patients identified seven upregulated proteins, FIBA, FIBB, FIBG, C09, VWF, LBP, and PRG-4, which are associated with neuroinflammatory processes and have been previously linked to ALS. Notably, PRG-4 enrichment in EVs was observed for the first time in ALS patients, with increased expression in patients with normal cognitive function. This suggest that elevated PRG-4 levels in serum and EVs may serve as a potential biomarker for ALS progression and cognitive impairment. Moreover, the increase in PRG-4+ EVs seen in ALS patients with normal cognitive function may have significant therapeutic potential for treating several other conditions in addition to ALS, comprising traumatic brain injury [41]. This suggests a potential role for PRG-4+ EVs in promoting recovery and long-term cognitive resilience in various neurological conditions. 

A major limitation of our study is the small cohort size, which may result in trends rather than conclusive associations between ALS scores and PRG-4 levels. Further analysis in a larger cohort is needed to validate PRG-4+ EVs as a biomarker for ALS progression. Furthermore, better characterization of PRG-4+ EVs’ cargo can potentially uncover novel connections between ALS and PRG-4. Elucidating PRG-4’s role in ALS pathophysiology could offer novel insights into disease mechanisms and identify new therapeutic targets, ultimately advancing ALS management.

## Figures and Tables

**Table 1:** Clinical characteristics of ALS patients. Q1 (baseline); Q3 (evaluation after 12 months).

	ALS N = 61
	N (%)
Gender	
Male	39 (63.93%)
Female	22 (36.07%)
Form of the Disease	
Spinal	41 (67.21%)
Bulbar	20 (32.79%)
Phenotype	
Classic	32 (52.46%)
Bulbar	20 (32.79%)
PUMN	6 (9.84%)
Respiratory	3 (4.92%)
Cognitive Impairment	
Normal	39 (63.93%)
ALS-ci	20 (32.79%)
ALS-bi	1 (1.64%)
FTD	1 (1.64%)
Progression	
Slow	34 (55.74%)
Fast	27 (44.26%)
Age at blood collection, mean ± SD)	62.88 ± 12.60
Average monthly change in FVC, median (Q1–Q3)	−1.93 (−3.78; −1.00)
Average monthly change in BMI, median (Q1–Q3)	−0.02 (−0.16; 0.15)
Average monthly change in ALFRSR, median (Q1–Q3)	−0.60 (−1.31; −0.27)

**Table 2:** The 32 proteins in circulating EVs identified in the discovery phase, which exhibited changes in ALS patients compared to HCs. *p* values below 0.05 and fold change > 1.2 were considered significant.

Protein Name	Fold Change (FC)	*p* Value
P20742|PZP_HUMAN Pregnancy zone protein	3.97	0.01
Q8TDD5|MCLN3_HUMAN Mucolipin-3	2.00	0.02
P18428|LBP_HUMAN Lipopolysaccharide-binding protein	1.84	0.02
Q92954|PRG4_HUMAN Proteoglycan 4	1.72	0.02
P04275|VWF_HUMAN von Willebrand factor	1.58	0.01
P02679|FIBG_HUMAN Fibrinogen gamma chain	1.45	0.01
O00187|MASP2_HUMAN Mannan-binding lectin serine protease 2	1.44	0.02
P22792|CPN2_HUMAN Carboxypeptidase N subunit 2	1.41	0.03
P02675|FIBB_HUMAN Fibrinogen beta chain	1.41	0.01
P01023|A2MG_HUMAN Alpha-2-macroglobulin	1.39	0.02
P02751|FINC_HUMAN Fibronectin	1.38	0.01
P04003|C4BPA_HUMAN C4b-binding protein alpha chain	1.37	0.01
P02671|FIBA_HUMAN Fibrinogen alpha chain	1.36	0.02
P01024|CO3_HUMAN Complement C3	1.34	0.005
P02748|CO9_HUMAN Complement component C9	1.33	0.02
P01031|CO5_HUMAN Complement C5	1.31	0.02
P07225|PROS_HUMAN Vitamin K-dependent protein S	1.31	0.04
P02747|C1QC_HUMAN Complement C1q subcomponent subunit C	1.27	0.04
P01009|A1AT_HUMAN Alpha-1-antitrypsin	1.26	0.01
P05156|CFAI_HUMAN Complement factor I	1.23	0.01
P05452|TETN_HUMAN Tetranectin	0.79	0.01
P04004|VTNC_HUMAN Vitronectin	0.79	0.02
P01344|IGF2_HUMAN Insulin-like growth factor II	0.78	0.03
P06396|GELS_HUMAN Gelsolin	0.72	0.002
P25311|ZA2G_HUMAN Zinc-alpha-2-glycoprotein	0.70	0.01
P05090|APOD_HUMAN Apolipoprotein D	0.64	0.004
P07477|TRY1_HUMAN Trypsin-1	0.39	0.01
Q12805|FBLN3_HUMAN EGF-containing fibulin-like extracellular matrix protein 1	0.33	0.03
P15924|DESP_HUMAN Desmoplakin	0.29	0.02
Q08188|TGM3_HUMAN Protein-glutamine gamma-glutamyltransferase E	0.25	0.04
P14923|PLAK_HUMAN Junction plakoglobin	0.21	0.04
P17931|LEG3_HUMAN Galectin-3	0.11	0.02

**Table 3:** Twenty proteins differentially expressed in ALS patients and HCs found in the validation phase. Each row represents a different protein identified in the study, with associated statistical significance and the magnitude of change in expression compared to control conditions. In bold are shown proteins found in both phases. *p* values below 0.05 and fold change > 1.2 were considered significant.

Protein Name	Fold Change (FC)	*p* Value
**Q92954|PRG4_HUMAN Proteoglycan 4**	**2.07**	**1.49 × 10^−5^**
O60306|AQR_HUMAN Intron-binding protein aquarius	1.94	0.01
**P04275|VWF_HUMAN von Willebrand factor**	**1.75**	**0.002**
**P02671|FIBA_HUMAN Fibrinogen alpha chain**	**1.75**	**0.001**
Q13103|SPP24_HUMAN Secreted phosphoprotein 24	1.74	0.009
Q9BWP8|COL11_HUMAN Collectin-11	1.66	0.02
Q9HDC9|APMAP_HUMAN Adipocyte plasma membrane-associated protein	1.63	0.04
P11169|GTR3_HUMAN Solute carrier family 2, facilitated glucose transporter member 3	1.60	0.02
P61626|LYSC_HUMAN Lysozyme C	1.58	0.043
**P02675|FIBB_HUMAN Fibrinogen beta chain**	**1.58**	**0.005**
**P18428|LBP_HUMAN Lipopolysaccharide-binding protein**	**1.56**	**0.001**
P01833|PIGR_HUMAN Polymeric immunoglobulin receptor	1.54	0.06
**P02679|FIBG_HUMAN Fibrinogen gamma chain**	**1.50**	**0.007**
P01034|CYTC_HUMAN Cystatin-C	1.47	0.02
P02786|TFR1_HUMAN Transferrin receptor protein 1	1.45	0.04
**P02748|CO9_HUMAN Complement component C9**	**1.41**	**0.001**
Q6UXB8|PI16_HUMAN Peptidase inhibitor 16	1.34	0.04
Q06033|ITIH3_HUMAN Inter-alpha-trypsin inhibitor heavy chain H3	1.28	0.02
P02763|A1AG1_HUMAN Alpha-1-acid glycoprotein 1	1.26	0.04
O00391|QSOX1_HUMAN Sulfhydryl oxidase 1	1.23	0.01

## Data Availability

The authors declare that the data supporting the findings of this study are included within the article and are available from the corresponding author upon reasonable request.

## Supplementary Materials

The following supporting information can be downloaded at: https://www.mdpi.com/article/10.3390/biom14060727/s1, Figure S1. Correlation between EVs concentration age and gender in HC (A, C) and ALS patients (B, D); Figure S2. Heatmap depicting the abundance of modulated proteins detected by proteomic analysis in the discovery phase in 12 HC and 12 ALS; Figure S3. Heatmap showing the prevalence of common proteins identified through proteomic analysis in the validation phase between 38 ALS patients and 24 HC; Figure S4. Correlation between PRG-4 protein abundance and age (A) and gender (B) in HC. Table S1. FIBA, FIBB, FIBG, C09, VWF, LBP and PRG-4 proteins associated with gender of ALS patients, Table S2. FIBA, FIBB, FIBG, C09, VWF, LBP and PRG-4 proteins correlated with the form of the disease, Table S3. FIBA, FIBB, FIBG, C09, VWF, LBP and PRG-4 proteins associated with the phenotype, Table S4. FIBA, FIBB, FIBG, C09, VWF, LBP, PRG-4 proteins linked with the progression of the disease, Table S5. FIBA, FIBB, FIBG, C09, VWF, LBP, PRG-4 proteins correlation with ALSFRS-R score and FVC at baseline and Table S6. FIBA, FIBB, FIBG, C09, VWF, LBP, PRG-4 proteins associated with cognitive behavior.
