# Supplementary material for: Shotgun Proteomics Links Proteoglycan-4+ Extracellular Vesicles to Cognitive Protection in Amyotrophic Lateral Sclerosis
Source: Biomolecules. 2024 Jun 19;14(6):727. doi: 10.3390/biom14060727 (PMC11202157; doi:10.3390/biom14060727)
Supplement: Supplementary file 1 [file biomolecules-14-00727-s001.zip › biomolecules-3024508-supplementary.pdf]

Supplementary Materials

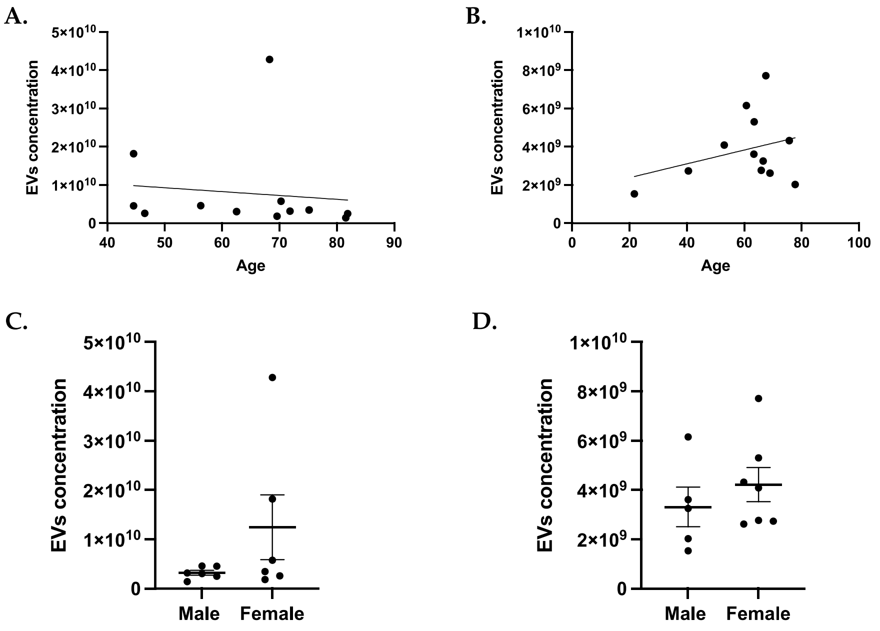

Figure S1. Correlation between EVs concentration age and gender in HC (A, C) and ALS patients (B, D).

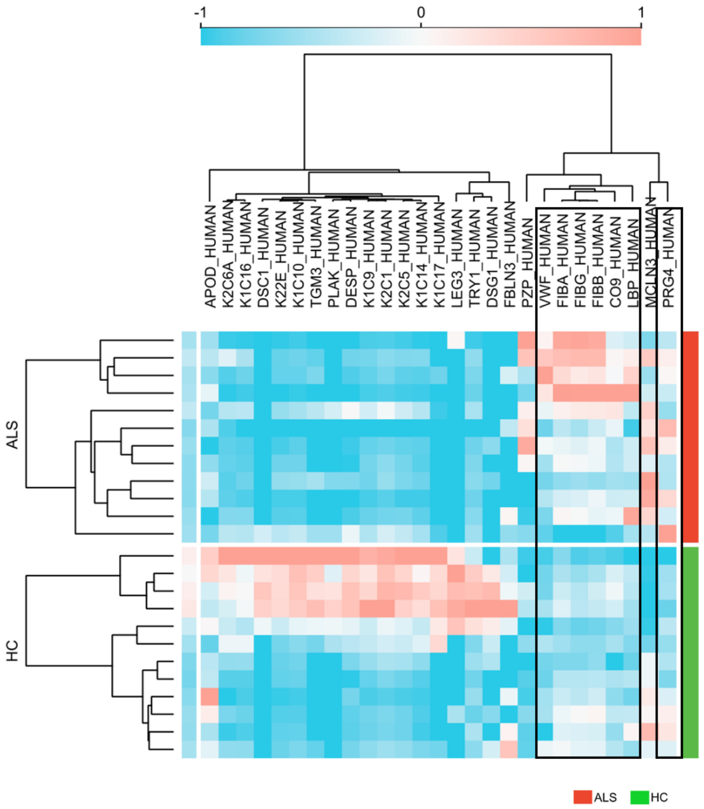

Figure S2. Heatmap depicting the abundance of modulated proteins detected by proteomic analysis in the discovery phase in 12 HC and 12 ALS.

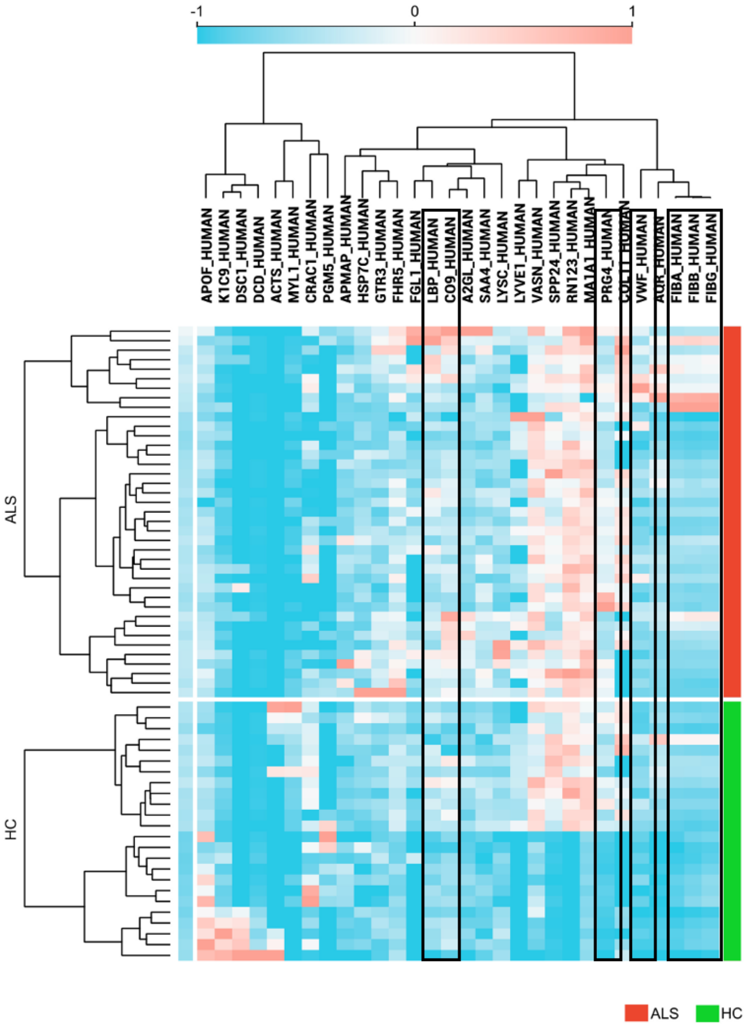

Figure S3. Heatmap showing the prevalence of common proteins identified through proteomic analysis in the validation phase between 38 ALS patients and 24 HC.

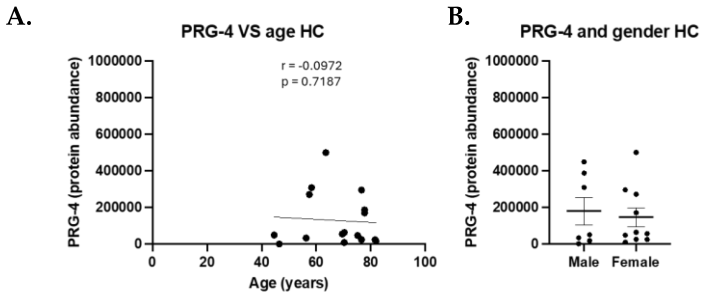

Figure S4. Correlation between PRG-4 protein abundance and age (A) and gender (B) in HC.

Table S1. FIBA, FIBB, FIBG, C09, VWF, LBP and PRG-4 proteins associated with gender of ALS patients.

	Gender			
	**Male N=28**	**Female N=11**		
	**Median (Q1-Q3)**	**Median (Q1-Q3)**	**Fold change**	**p-value**
**FIBA**	689925532 (513365125-835853272)	701948265 (611861635-1294889258)	1.017	0.2863
**FIBB**	637921473 (487995416-812353113)	653233065 (581384287-1182131866)	1.024	0.3307
**FIBG**	702519816 (553954706-899734323)	750998921(638651795-1293927330)	1.069	0.2724
**C09**	355993 (241369-397196)	286544 (236557-401029)	0.084	0.7939
**VWF**	15931121 (12303324-24397482)	17095378 (16031783-26737916)	1.073	0.2459
**LBP**	495971 (380157-637498)	482376 (385024-631125)	0.972	0,8416
**PRG-4**	355993 (241369 -397196)	286544 (236557-401029)	0.805	0.6556

Table S2. FIBA, FIBB, FIBG, C09, VWF, LBP and PRG-4 proteins correlated with the form of the disease.

	Form of the disease			
	**Spinal N=25**	**Bulbar N=13**		
	**Median (Q1-Q3)**	**Median (Q1-Q3)**	**Fold change**	**p-value**
**FIBA**	795774510 (590033058-1282900690)	689925532 (511354614-801826674)	0.867	0.1939
**FIBB**	761861366 (548663696-1181451417)	624010520 (488849489-716075064)	0.819	0.1566
**FIBG**	833640151 (622784906-1301449711)	668963264 (553100119-797464303)	0.802	0.1566
**C09**	10437918.41 (8700148.58-16145169.2)	11065405.68 (8270720.36-13349739.78)	1.060	0.9027
**VWF**	17721259.03 (14115963.75-24451899.11)	16384911.49 (12257706.91-24849290.69)	0.925	0.9513
**LBP**	477006.6 (383917.65-642178.48)	499983.12 (406051.85-624304.73)	1.048	0.5830
**PRG-4**	281055.14 (239088.52-390113.29)	359148.77 (241284.61-394927.76)	1.278	0.8069

Table S3. FIBA, FIBB, FIBG, C09, VWF, LBP and PRG-4 proteins associated with the phenotype.

	Phenotype			
	Classic N=20	Bulbar N=14		
	Median (Q1-Q3)	Median (Q1-Q3)	Fold change	p-value
**FIBA**	795774510 (590033058-1282900690)	689925532 (511354614-801826674)	0.867	0.1209
**FIBB**	761861366 (548663696-1181451417)	624010520 (488849489-716075064)	0.819	0.0926
**FIBG**	833640151 (622784906-1301449711)	668963264 (553100119-797464303)	0.802	0.0752
**C09**	10437918.41 (8700148.58-16145169.2)	11065405.68 (8270720.36-13349739.78)	1.060	0.7680
**VWF**	17721259.03 (14115963.75-24451899.11)	16384911.49 (12257706.91-24849290.69)	0.925	0.4998
**LBP**	477006.6 (383917.65-642178.48)	499983.12 (406051.85-624304.73)	1.048	0.8486
**PRG-4**	281055.14 (239088.52-390113.29)	359148.77 (241284.61-394927.76)	1.278	0.5219

Table S4. FIBA, FIBB, FIBG, C09, VWF, LBP and PRG-4 proteins linked with the progression of the disease.

	Disease progression			
	Slow N=20	Fast N=18		
	Median (Q1-Q3)	Median (Q1-Q3)	Fold change	p-value
**FIBA**	767566909 (552600452-1031035700)	672707938 (588782201-898711919)	0.876	0.7386
**FIBB**	710373470 (503946529-949441556)	596130066 (501809999-818892826)	0.839	0.5721
**FIBG**	765790248 (600359200-1034245946)	688133005 (590351323-884897833)	0.899	0.4783
**C09**	11570746.41 (9106040.84-16145169.2)	10501325.5 (8131113.39-13349739.78)	0.908	0.5147
**VWF**	16200306.55 (13395927.02-20743519.59)	17119457.94 (12440176.35-31192837.5)	1.057	0.4783
**LBP**	517561.43 (418425.67-633100.04)	470747.65 (371525.74-631124.83)	0.910	0.4783
**PRG-4**	339361.39 (212936.71-379616.54)	379719.68 (243939.84-415608.82)	1.119	0.1563

Table S5. FIBA, FIBB, FIBG, C09, VWF, LBP and PRG-4 correlated with ALSFRS-R score and FVC% at the baseline. P values below 0.05 were considered significant.

	**ALSFRS-R Baseline**	**FVC Baseline**
	r (p-value)	r (p-value)
**FIBA**	-0.1103 (0.5096)	-0.08154 (0.6573)
**FIBB**	-0.1495 (0.3705)	-0.1247 (0.4964)
**FIBG**	-0.1577 (0.3444)	-0.1137 (0.5356)
**C09**	-0.4774 (0.0024)	-0.4083 (0.0203)
**VWF**	-0.3469 (0.0329)	-0.2211 (0.2239)
**LBP**	0.1026 (0.9513)	-0.2144 (0.2387)
**PRG-4**	0.2019 (0.2241)	0.1721 (0.3462)

Table S6. Association analysis of proteins expressed as abundance found in both phase (discovery and validation) with the cognitive-behavioral profile in ALS patients. FIBA, FIBB, FIBG, von VWF, C09, PRG-4 and LBP.

	**Cognitive behavior**			
	**Normal N=25**	**ALS-ci N=12**	**Fold change**	**p-value**
	Median (Q1-Q3)	Median (Q1-Q3)		
**FIBA**	7.6e10^8 (5.9e10^8-9.2e10^8)	6.4e10^8 (5.1e10^8-8.2e10^8)	0.841	0.5957
**FIBB**	7.1e10^8 (5.2e10^8-8.7e10^8)	6.2e10^8 (4.6e10^8-7.7e10^8)	0.877	0.7113
**BIBG**	7.6e10^8 (6.1e10^8-9.7e10^8)	7.0e10^8 (5.2e10^8-8.7e10^8)	0.921	0.6875
**C09**	1.1e10^7 (9e10^6-1.2e10^7)	1.3e10^7 (8.8e10^6-1.6e10^7)	1.204	0.3782
**VWF**	1.8e10^7 (1.3e10^7-2.3e10^7)	1.4e10^7 (1.2e10^7-2.2e10^7)	0.844	0.2843
**LBP**	5.1e10^5 (3.8e10^5-6.4e10^5)	4.8e10^5 (4.1 e10^5-6e10^5)	0.962	0.8847
**PRG-4**	3.7e10^5 (2.7e10^5-4.2e10^5)	2.6e10^5 (2.1e10^5-3.4e10^5)	0.696	0.0208
